# Interdisciplinary public engagement: untapped potential?

**DOI:** 10.1242/bio.060108

**Published:** 2024-03-25

**Authors:** Janet Stott, Catarina C. Vicente

**Affiliations:** ^1^ Reuben College, University of Oxford, Reuben College, OX1 3QP, Oxford, UK; ^2^ Oxford University Museum of Natural History, Dunn School, OX1 3RE, Oxford, UK; ^3^ Sir William Dunn School of Pathology, University of Oxford, Museum, OX1 3PW, Oxford, UK

**Keywords:** Public engagement, Outreach, Interdisciplinary, Public understanding of science

## Abstract

Public engagement projects developed by university-based academics tend to focus on specific academic topics. Yet, the problems and topics that our audiences want to engage with are broad, challenging, and can't be explained or solved by a single academic subject or expertise. In this article, we capitalise on our experience working with academics at the University of Oxford, and a workshop for public engagement professionals that we co-organised with the National Coordinating Centre for Public Engagement, to advocate for a novel approach: interdisciplinary public engagement (public engagement projects that bring together academics from several academic disciplines). We consider the potential benefits and the challenges of this approach and provide examples of how it is starting to be explored.

## Teaser

**Summary:** We propose that interdisciplinary public engagement (public engagement projects that bring together academics from several academic disciplines) is a novel and potentially mutually beneficial approach to engage the public.

## The problem

Public engagement initiatives developed by university-based academics tend to focus on particular academic subjects: If you are a biologist, you will engage on biology-specific topics; if a chemist, on chemistry-specific topics. There are many reasons for this. In academic research, you are encouraged to specialise, to focus on a specific aspect of a discipline. It is therefore natural to develop public engagement with the same focus. Secondly, departments (which are often subject focussed) are the main organisational unit of universities (Villa-Komaroff, 2019). From our experience, many local professional public engagement support and initiatives tend therefore to be organised around these administrative units. Departments are also the main beneficiary units of the Research Excellence Framework (REF), a major motivator for engagement and impact initiatives. This siloing is further emphasised by the fact that most universities are further organised into broader administrative units that separate STEM subjects from the humanities, social sciences and the arts (Skorton and Bear, 2018). Thirdly, most existing sources of funding for public engagement activities are also subject focussed, either because they are provided by departments, by research funders or scientific societies (see, for example, https://www.publicengagement.ac.uk/funding-opportunities).

Yet, for the audiences that these projects hope to engage with, individual academic subjects are meaningless. If your target audience are cancer patients, it is irrelevant for them whether you are a biologist, a chemist, a clinician, a physicist or an engineer. The questions that they ask will be broad, far-reaching and based on personal experience: they cannot be constrained within the boundaries of a single discipline. What matters for this audience is how research (in its broadest definition) can provide knowledge and solutions for those affected. The same principles could be applied to any other target audience or topic of interest. Several surveys and reports on public perceptions of research support this view. For example, CaSE's 2022–2023 report on public perception of research and development (R&D) in the UK noted as a major trend that ‘linking R&D to the problem it's solving helps it feel relevant to more people’ (CaSE, 2023). A benchmark study on public attitudes to science in the USA, conducted by Science Counts in 2015, also highlighted the importance of presenting individual and community benefits to engage successful with audiences (ScienceCounts, 2015). It is clear that our audience's interests are focused on themes or problems that affect society at large, or themselves personally, not the specific academic subject of the person leading the public engagement activity.

We are Official Fellows in Public Engagement with Research at Reuben College, University of Oxford. Reuben College is a community of academics and students from across a variety of departments and academic subjects at Oxford. In this unique context, we have become interested in exploring the role of interdisciplinary public engagement in the existing UK public engagement landscape, and how this approach may add value to our efforts to engage. Our experience at Reuben (Box A) is just one of the ways in which we and others are trying to either capitalise on existing interdisciplinary structures or create new ones (Boxes B and C) to explore this new concept and, eventually, encourage interdisciplinary collaborations in public engagement. In April 2021, we collaborated with the National Coordinating Centre for Public Engagement to run a joint workshop for UK-based public engagement professionals, where we explored the concept of interdisciplinary public engagement. The views and opinions generously shared by the participants of that workshop, as well as our own experiences at Reuben fostering this specific flavour of public engagement, are the basis for this article. This is not yet a tried and tested approach. Rather, we propose it here as a helpful conceptual framework that we, and others, can explore going further and on which we hope to be able to provide evaluation data on a later date.

**Box A. Exploiting existing interdisciplinary structures: the Reuben College example**

At the University of Oxford, where we are based, there are formalised structures that cross subject areas called colleges. While not unique to Oxford, the collegiate system is unusual in historically preceding the concept of the department. Colleges are communities that provide a teaching, pastoral and social structure (as well as a physical space) for students and academics across the spectrum of disciplines, from the humanities to sciences and mathematics. Students and academics are both affiliated with a department and with a college, and hence are automatically part of an interdisciplinary community alongside the conventional departmental affiliation that would be expected in most universities.

In our experience, public engagement is not traditionally seen as part of the life or mission of colleges. No doubt discussions between students and academics on public engagement take place, but public engagement support and opportunities are generally seen as something that is the responsibility of departments. Reuben College is unique among colleges in having a commitment to public engagement at the core of its vision and mission, and to have appointed two fellows (the authors of this article) specifically on their expertise in public engagement. The college is exploring ways in which it can be a platform for public engagement, and in particular complement public engagement that already exists at the University of Oxford.

This naturally interdisciplinary community is a great place to experiment with the concept of interdisciplinary public engagement. At its early stages, this is being explored through various initiatives, including a regular academic seminar and discussion series that engage the community from interdisciplinary perspectives, and the establishment of a student public engagement fund primarily supporting interdisciplinary projects.

**Fig. 1.**
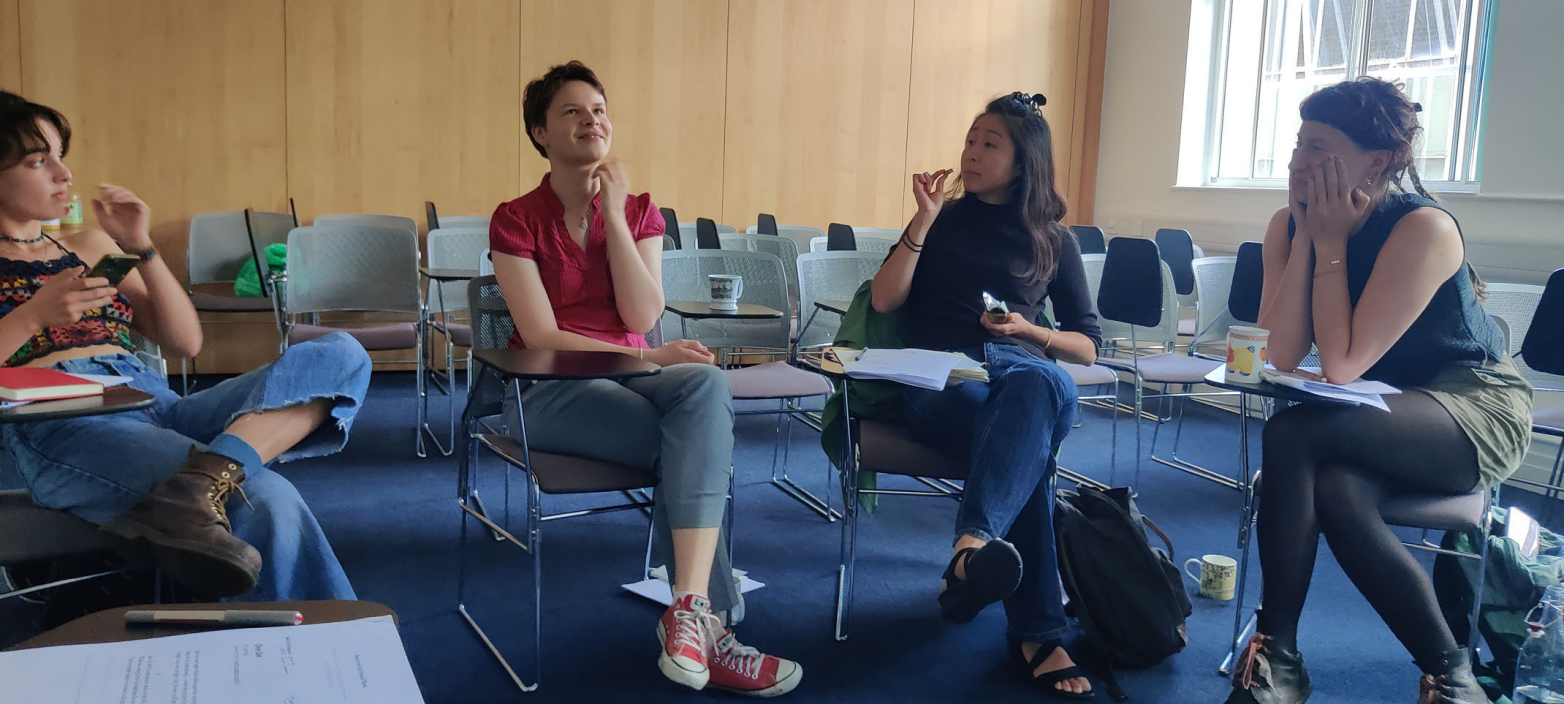
Student-led poetry workshop on environmental themes, supported by a Reuben College Public Engagement Award.

Catarina Vicente and Janet Stott (Official Fellows in Public Engagement with Research, Reuben College) (Fig. 1).

**Box B. Creating an over-arching university public engagement structure that is department-blind: The Warwick Institute of Engagement example**

Warwick Institute of Engagement (WIE) is different from a standard public engagement team in three ways. First WIE's core team is composed of both professional services public engagement specialists and academics (with a part-time buy-out from their departmental roles). Second, WIE recruited over 150 fellows from across professional services teams, academic departments and its student body to work, through a series of Learning Circles dedicated to different aspects of public engagement (e.g. inclusive PE; the pedagogy of PE), in tandem with the core team to pool wide, interdisciplinary, experience and expertise from across all sectors, disciplines and career stages, feeding into the Institute's strategic programmes. Third, WIE also recruited 12 Regional Fellows, community leaders within different fields in Coventry and Warwickshire, who regularly feed in their experience, expertise and understanding to the development of WIE projects.

Using this framework, which taps into all areas of the university at all levels, alongside our regional fellows, WIE has focused both on building capacity and expertise across the university for public engagement, and then creating the right opportunities for students and staff to engage in diverse ways regionally and nationally. In particular, we developed the Resonate event programme. Resonate events vary in nature, from specific talks on specific (disciplinary) topics to our regular ‘Resonate Lates’, which are interdisciplinary discussions on wider interdisciplinary topics (e.g. AI; mental health); to topics and themes suggested by our community partners, to which we bring a range of research angles; through to our annual Resonate on-campus festival, where over 3 days the widest possible range of topics are tackled for audiences who sample far and wide to pursue an interest or expose themselves to something new.

Michael Scott (Director of the Warwick Institute of Engagement)

**Box C. Capitalising on common spaces to promote interdisciplinary public engagement: the Oxford University Museum of Natural History example.**

As a university museum, the Oxford University Museum of Natural History is in an ideal position to combine its expertise in engagement with diverse public audiences with the research power of the University of Oxford. Since 2014 the museum has programmed a series of contemporary science and society exhibitions which aim to engage museum visitors with contemporary science in an accessible way, creating a space for debate and discussion. The exhibitions are interdisciplinary and based around themes relating to the science of the natural environment that have societal relevance. The exhibitions team works with a group of researchers from different disciplines to shape the narrative and content of the exhibition in a collaborative process. Exhibitions typically run for 9 months and receive over 200,000 visitors.

For example: ‘Bacterial World’ sought to tell the untold story of bacteria - how they shaped the Earth's past, and continue to wield huge influence over us all. The core exhibition team included researchers from departments of Biochemistry, Earth Sciences, Zoology, Geography and the Environment, Plant Sciences and Pathology, working together to create an interdisciplinary exhibition. In addition to the core exhibition team, the museum worked with a further 96 researchers in the extensive exhibition programme (39 varied events from a comedy night, to a board games event, lectures, and sauerkraut and sourdough workshops) providing opportunities for a wide range of researchers to engage with the public in a supported way. In addition to the science content developed for the exhibition, the museum includes contemporary art installations within the exhibition, to broaden the engagement of audiences with the research content.

Other examples of interdisciplinary exhibitions developed by OUMNH include Settlers: Genetics, geography and the peopling of the Britain, Brain Diaries, First Animals, Meat the Future, Connected Planet and the current exhibition, Fair Water.

**Fig. 2.**
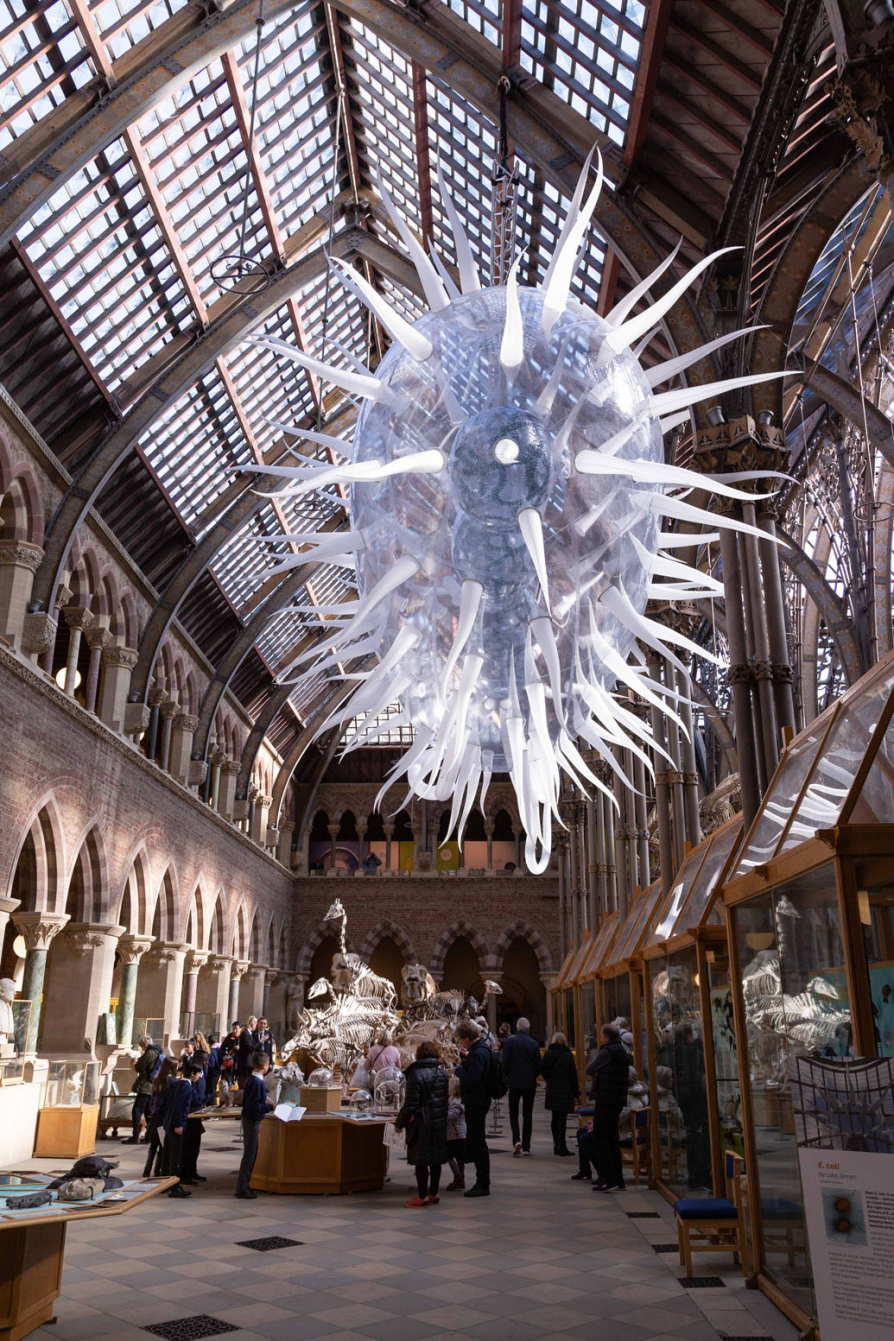
Artwork as part of an interdisciplinary exhibition at the Oxford University Museum of Natural History.

**Fig. 3.**
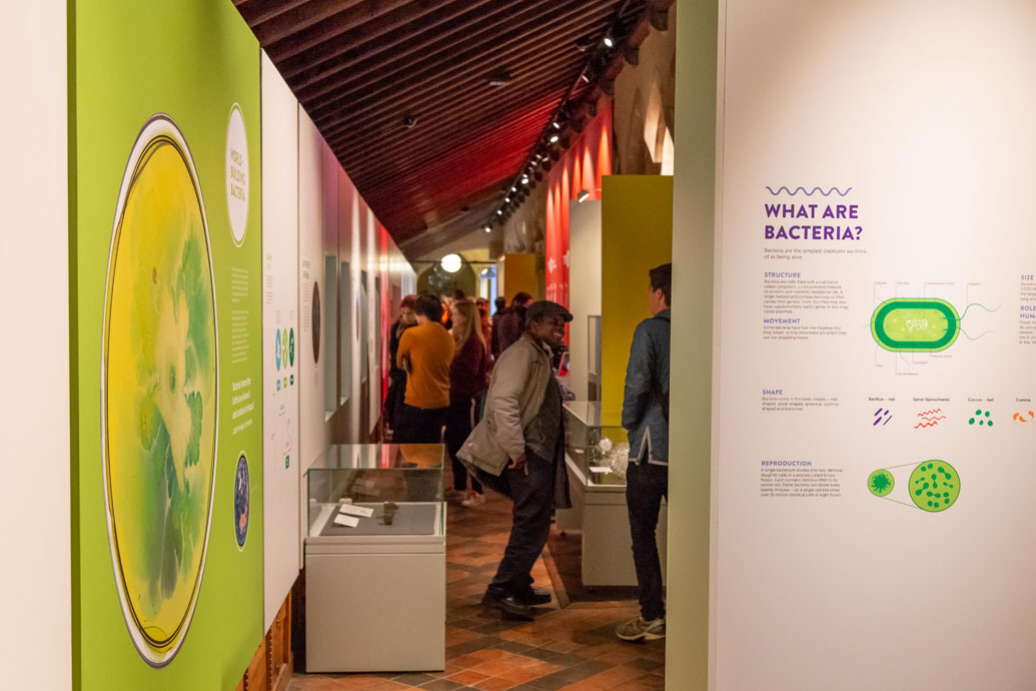
Bacterial World exhibition at Oxford University Museum of Natural History.

Janet Stott (Deputy Director Oxford University Museum of Natural History) (Figs 2 and 3).

## Definition

In this piece, we want to focus on interdisciplinary public engagement, defined as public-engagement initiatives that bring together researchers who identify with different academic subjects, to produce an activity that provides a holistic view of a subject/challenge/problem of interest to an audience. In our view, it is important that the sum is greater than its parts, i.e. that the resulting project provides a broad, connected view of the topic, rather than just separate (subject-specific) perspectives on the same topic. Though sometimes difficult, this would ensure that both researchers and publics can reap the full rewards of this approach (see ‘The benefits’, below).

To fully understand our definition, it is helpful to consider each of its constituent terms separately. Importantly, we are specifically talking about public engagement, and not outreach. The use of the word ‘engagement’ reflects the ambition that the activity will promote a two-way interaction between researchers and communities (see www.publicengagement.ac.uk/introducing-public-engagement). The term ‘outreach’ suggests a one-way flow of information, almost invariably from the researcher to the public, and hence could be misunderstood as simply broadcast. Though we acknowledge that there is a range of potential ways of interacting with publics between these two extremes, our aspiration for interdisciplinary public engagement is that it may be an ideal conduit for true engagement.

In our definition, ‘disciplines’ in interdisciplinarity refer to academic disciplines, as would be recognised in a university context. We do not wish to limit this definition to STEM subjects though. In our view, the most valuable interdisciplinary public engagement is that where STEM, humanities, social sciences and the arts cross paths. There are alternative interpretations of ‘discipline’, outside the scope of this article. For example, it is valid to consider other forms of knowledge (such as lived experience, see, for example, WHO, 2023) as equal partners in interdisciplinary public engagement. We are limiting ourselves in this article to academic disciplines because our experience lies primarily in a university context. However, we acknowledge that, by focusing on this narrower definition, we risk reinforcing stereotypes on the relative value of different types of knowledge.

It is also useful to consider what we mean by ‘inter’ disciplinarity, as opposed to multi or intra-disciplinarity. It can be unhelpful to spend too long considering exactly what these different terms mean. Our intention is to encourage projects in which academics from different disciplines work together to produce a public engagement output. That output must genuinely involve the collaboration of those different parties, in such a way that the outcome is greater than the sum of its parts, rather than only providing separate (academic discipline-focused) perspectives on a common topic. Imagine that you wish to develop a tabletop public-engagement activity in a science festival on the topic of climate change. A multi-disciplinary approach could manifest itself in a series of different stations, each led by academics in a different field. A chemist might have a booth talking about the effect of certain chemical releases in the atmosphere and how these impact on climate change; a biologist might discuss the impacts of climate change on local ecology and species survival; an engineer on technical solutions to remove carbon from the atmosphere; and an anthropologist on the relative impact of climate change on different cultures and societies. A truly interdisciplinary public engagement approach would present a single booth on climate change that would not present each of these topics as separate. Instead, all players would input on content to approach the problem and the solutions holistically, providing a full view of climate change.

It is important for us to distinguish between interdisciplinary public engagement (this definition) and public engagement on interdisciplinary research. The latter will follow naturally from interdisciplinary research projects, and with increasing focus on this type of research, we expect it to become increasingly common. However, with academic research still primarily anchored on traditional academic subjects, it seems unlikely that public engagement on interdisciplinary research will be dominant in the near to middle future. Interdisciplinary public engagement should be within reach of all researchers, regardless of whether they are part of interdisciplinary research collaborations, with benefits for both researchers and the public.

## The benefits

Any good quality two-way public engagement should benefit both researchers and the public. However, we believe that interdisciplinary public engagement can bring unique benefits.

Placing audience at the centre is key to any public engagement activity. This can only be achieved if the interests, experiences and realities of that target audience drive the engagement. For audiences, the main potential benefit of interdisciplinary public engagement is its ability to provide a genuinely holistic understanding of the theme, topic or challenge. It can also potentially promote a more engaged form of dialogue. The target public will be able to ask and answer questions that cut across academic disciplines, rather than being faced with the all-too-common answer ‘that is outside the remit of this activity’. Additionally, the target public would potentially be provided with a more realistic view of the interconnectedness of the research process, and how even the most focused research projects contribute to the broader topic and to real world solutions and understanding. Ultimately, this can lead to benefits to researchers themselves, as it provides a more realistic view of how research hopes to impact the real world, and therefore may increase public support for discovery research.

There are also other potential benefits for academics. We believe that interdisciplinary public engagement can provide a unique public engagement training opportunity. By working with researchers from other fields, no individual academic participant will be familiar on all the topics involved. Therefore, each participant will both be a specialist (on some of the concepts) and an audience member (on other concepts). By playing both roles (rather than just being ‘the specialist’ as is the case in more traditional public engagement), as well as working with peers (albeit from different subjects), interdisciplinary public engagement will allow researchers to practice one of the simplest, yet hardest, principles of good public engagement: to assume 100% intelligence and 0% knowledge.

Another benefit for academics is a new way of looking at their own research, and to put it in the context of the bigger picture. This can be generated in two ways. First, by interacting with publics outside the constraints of discipline, which will inevitably generate unique questions that will encourage the researcher to think more broadly about their research. It will also encourage interactions with researchers from other fields. This will not only generate the same broad perspectives, but may even foster new interdisciplinary research collaborations, by bringing together researchers from different fields that might not otherwise come across each other. Oxford immunologist Professor Hal Drakesmith experienced the benefits of interdisciplinary public engagement in his research first hand: ‘We participated in a public engagement event during the 2019 Oxford Science and Ideas Festival that brought together biologists, geologists, archaeologists and anthropologists to discuss the science of iron from a variety of perspectives. This event cemented an interdisciplinary research project with fellow participant Professor Jon Wade, from the department of Earth Sciences, which continues to this day with a joint PhD student, joint grants and already one joint publication (Wade, et al., 2021)’.

## The challenges

The most obvious challenge of interdisciplinary public engagement is also the one faced by interdisciplinary research. Different fields bring with them different language and nomenclature, and also very different views of what outputs are desirable or what success should look like (see, for example, Pellmar et al., 2000). Breaking these barriers to produce a joint project requires willingness to go outside one's comfort zone, to take time to listen and engage in genuine conversation. Ultimately, to be willing to compromise and work together. Though of great benefit both personally and academically, these barriers can be limiting when participants are under wider work pressures and have limited time to dedicate to public engagement.

Another challenge that may impact the outcomes of an interdisciplinary public engagement project is the unconscious perception, even within academic fields, that certain forms of knowledge or expertise are more valuable than others. For example, science/art collaborations have flourished in the last few years (Gewin, 2021; Chappell and Muglia, 2023), with many beautiful and creative outcomes. Yet, many of these struggle to be genuine partnerships of equals, and often artists are expected to embed themselves into the scientific process and ‘learn’ from their STEM colleagues in order to produce an art piece, without there being an expectation that the scientists should go through an equal process to understand and reflect on the processes and expertise of the artists. A willingness to work together as equals is essential to achieve true interdisciplinary public engagement.

Some of the other challenges that may impact interdisciplinary public engagement are institutional or structural. With limited time to dedicate to public engagement, it is not surprising that most public engagement projects happen locally at a departmental level. With most academics siloed into their own departments, how can we foster public engagement collaborations involving academics from different subjects that do not already work together on a collaborative research project? There are ways in which this can be achieved, either by taking advantage of existing multi-disciplinary networks (see Box A) or efforts to cross departmental boundaries (see Boxes B and C). The initiation energy for these collaborations requires both commitment from researchers, but also funding and resources to foster and support these properly.

More broadly, interdisciplinary public engagement will face the same challenges and barriers as other forms of public engagement. From an academic perspective, these include lack of time, funding, etc, which are comprehensively covered elsewhere [see examples from the UK (Hamlyn et al., 2015), the USA (Calice et al., 2022) and Asia (Thai and Chambers, 2023)]. It is likely that some of these barriers will be exacerbated by the higher level of involvement and commitment that interdisciplinary public engagement would require, and this would have to be considered before choosing our proposed approach.

## Conclusion

In this article, we have made the case for how interdisciplinary public engagement could be a unique approach to engage with publics, with many potential benefits for both researchers and audiences. However, it is also clear that there are challenges, some of which apply to all engagement projects, others which are specific to this type of engagement. While interdisciplinary public engagement is unlikely to replace other forms of engagement, we believe that it has its place in the plethora of ways in which academics can engage with publics and with each other. In a changing world, where the biggest challenges cannot be solved by any single person or subject, interdisciplinary public engagement can become an increasingly relevant and powerful tool in the engagement toolbox available to researchers and public engagement professionals alike. Funders, scientific societies and departments can play a role in encouraging this type of engagement by helping put in place the funding, resources and expertise to help it flourish.
